# The Guinea-Bissau Family of *Mycobacterium tuberculosis* Complex Revisited

**DOI:** 10.1371/journal.pone.0018601

**Published:** 2011-04-20

**Authors:** Ramona Groenheit, Solomon Ghebremichael, Jenny Svensson, Paulo Rabna, Raffaella Colombatti, Fabio Riccardi, David Couvin, Véronique Hill, Nalin Rastogi, Tuija Koivula, Gunilla Källenius

**Affiliations:** 1 Department of Preparedness, Swedish Institute for Infectious Disease Control, Solna, Sweden; 2 Department of Microbiology, Tumor and Cell Biology, Karolinska Institutet, Stockholm, Sweden; 3 Laboratório Nacional de Saúde Pública, Bissau, Guinea-Bissau; 4 Bandim Health Project, Indepth Network, Bissau, Guinea-Bissau; 5 Hospital “Raoul Follereau”, Bissau, Guinea-Bissau; 6 Department of Pediatrics, Azienda Ospedaliera-Università di Padova, Padova, Italy; 7 Department of Public Health, University of “Tor Vergata”, Rome, Italy; 8 WHO Supranational TB Reference Laboratory, Tuberculosis and Mycobacteria Unit, Institut Pasteur de la Guadeloupe, Guadeloupe, France; 9 Department of Clinical Science and Education, Karolinska Institutet, Stockholm, Sweden; St. Petersburg Pasteur Institute, Russian Federation

## Abstract

The Guinea-Bissau family of strains is a unique group of the *Mycobacterium tuberculosis* complex that, although genotypically closely related, phenotypically demonstrates considerable heterogeneity. We have investigated 414 *M. tuberculosis* complex strains collected in Guinea-Bissau between 1989 and 2008 in order to further characterize the Guinea-Bissau family of strains. To determine the strain lineages present in the study sample, binary outcomes of spoligotyping were compared with spoligotypes existing in the international database SITVIT2. The major circulating *M. tuberculosis* clades ranked in the following order: AFRI (n = 195, 47.10%), Latin-American-Mediterranean (LAM) (n = 75, 18.12%), ill-defined T clade (n = 53, 12.8%), Haarlem (n = 37, 8.85%), East-African-Indian (EAI) (n = 25, 6.04%), Unknown (n = 12, 2.87%), Beijing (n = 7, 1.68%), X clade (n = 4, 0.96%), Manu (n = 4, 0.97%), CAS (n = 2, 0.48%). Two strains of the LAM clade isolated in 2007 belonged to the Cameroon family (SIT61). All AFRI isolates except one belonged to the Guinea-Bissau family, i.e. they have an AFRI_1 spoligotype pattern, they have a distinct RFLP pattern with low numbers of IS*6110* insertions, and they lack the regions of difference RD7, RD8, RD9 and RD10, RD701 and RD702. This profile classifies the Guinea-Bissau family, irrespective of phenotypic biovar, as part of the *M. africanum* West African 2 lineage, or the AFRI_1 sublineage according to the spoligtyping nomenclature. Guinea-Bissau family strains display a variation of biochemical traits classically used to differentiate *M. tuberculosis* from *M. bovis*. Yet, the differential expression of these biochemical traits was not related to any genes so far investigated (*narGHJI* and *pncA*). Guinea-Bissau has the highest prevalence of *M. africanum* recorded in the African continent, and the Guinea-Bissau family shows a high phylogeographical specificity for Western Africa, with Guinea-Bissau being the epicenter. Trends over time however indicate that this family of strains is waning in most parts of Western Africa, including Guinea-Bissau (p = 0.048).

## Introduction

Guinea-Bissau is a West African country with a population of approximately 1.6 million people. It is a country with one of the highest tuberculosis (TB) burdens in the world. In previous studies of *Mycobacterium tuberculosis* complex isolates from Guinea-Bissau, we identified a unique group of strains that we designated the Guinea-Bissau family of strains [1], [2]. Based on a genotypic analysis of a subset of Guinea-Bissau family isolates, and a comparison of known corresponding genes in mycobacteria outside the *M. tuberculosis* complex, we proposed that strains of the Guinea-Bissau family belong to a unique branch of the *M. tuberculosis* complex tree positioned between classical *M. tuberculosis* and classical *M. bovis* [2], clustering together within one of the *M. africanum* subtype I branches [3]. The most intriguing fact about the Guinea-Bissau family of strains is that, although genotypically closely related, they demonstrate a considerable heterogeneity of phenotypic traits [1], [2], with a variation in niacin production, pyrazinamide (PZA) resistance, and nitrate reductase activity, that are basic phenotypic criteria for differentiating *M. tuberculosis* and *M. bovis*. Yet, the differential expression of these biochemical traits by the Guinea-Bissau family of strains have not been related to any genes so far investigated [2].

Since the first *M. tuberculosis* strains from the late 1980s and early 1990s were isolated in Guinea-Bissau [1], [4] there has been a war in the country. This caused the TB program to dissolve, the Reference TB hospital was out of function and the TB laboratory at the Laboratório Nacional de Saúde Pública (LNSP), Bissau, was destroyed and no drug resistance surveillance could be performed. Culture of sputum samples has only recently been made possible again. We have now collected a new sample of *M. tuberculosis* complex isolates from patients with pulmonary TB and compared them to previously collected (published and unpublished) isolates in terms of genotype. Thus, we hereby reinvestigated *M. tuberculosis* complex clinical isolates from Guinea-Bissau (n = 414) collected between 1989 and 2008 with emphasis on the phylogeography, of the Guinea-Bissau family of strains, and their differential expression of biochemical traits.

## Materials and Methods

### Ethics Statement

The Research Committee at Karolinska Institutet exempted this retrospective study from review because it describes bacterial samples collected during the course of routine surveillance, and waived the need for consent due to the fact that the samples received could only be combined with the sex, age, and country of birth of the patients from which the strains were isolated.

### Patients

The isolates were collected consecutively as part of epidemiological TB studies in Guinea-Bissau. The patients had been referred to the Raoul Follereau Reference TB hospital in Bissau, Guinea-Bissau from various part of the country. The Hospital is the TB National Reference Center, therefore receives patients from the entire country, namely patients referred from regional hospitals or health centers and also patients coming by themselves. Patients come from the entire country (Bissau, Gabu, Bafata, Cacheu regions).

#### Study A

During 1989–1994, 229 isolates from the same number of patients with positive cultures for the *M. tuberculosis* complex were collected as part of an epidemiological TB study in Guinea-Bissau [1], [2], [4], [5], [6].

#### Study B

As part of a survey of drug resistance, a systematic collection of *M. tuberculosis* complex isolates was performed between 1994–1998. Approximately 700 sputum samples from the same number of patients with suspected pulmonary TB were collected at the Raoul Follereau Reference TB hospital. Positive culture for the *M. tuberculosis* complex was confirmed for 192 samples.

#### Study C

During 2006, 2007 and 2008 approximately 400 sputum samples from the same number of patients were collected. Out of these, 85 were culture positive for the *M. tuberculosis* complex.

### Mycobacterium tuberculosis complex isolates

Isolates included in study A, B and late part of study C were cultured at the LNSP as previously described [1]. Culture in the early part of study C was performed at the TB laboratory, Karolinska University Hospital, Stockholm, Sweden. All *M. tuberculosis* complex isolates were transported to the Reference TB laboratory at the Swedish Institute for Infectious Disease Control, Solna, Sweden, for further molecular characterization.

### Spoligotyping

Spoligotyping relied on the amplification of the polymorphic direct repeat region [7] to obtain hybridization patterns of the amplified DNA using multiple synthetic spacer oligonucleotides, which are covalently bound to a membrane (Isogen Bioscience BV, The Netherlands).

### Database comparison of spoligotypes

Spoligotypes in binary format were entered in the SITVIT2 database (Pasteur Institute of Guadeloupe), which is an updated version of the previously released SpolDB4 database [8]. Of 414 isolates 229 had been previously entered by us into the database, and 185 were added in conjunction with this study. At the time of the present study, SITVIT2 contained genotyping information on about 75,000 *M. tuberculosis* clinical isolates from 160 countries of origin. In this database, SIT (Spoligotype International Type) designates spoligotypes shared by two or more patient isolates, as opposed to “orphan” which designates patterns reported for a single isolate. Major phylogenetic clades were assigned according to signatures provided in SpolDB4, which defined 62 genetic lineages/sub-lineages. These include specific signatures for various *M. tuberculosis* complex species such as *M. bovis, M. microti, M. caprae, M. pinipedii,* and *M. africanum*, as well as rules defining major lineages/sublineages for *M. tuberculosis* sensu stricto. These include the Beijing clade, the Central Asian (CAS) clade and two sublineages, the East African-Indian (EAI) clade and nine sublineages, the Haarlem (H) clade and three sublineages, the Latin American-Mediterranean (LAM) clade and 12 sublineages, the “Manu” family and three sublineages, the S clade, the IS*6110*–low-banding X clade and four sublineages, and an ill-defined T clade with five sublineages.

### IS*6110* RFLP analyses

A standardized method for IS*6110* RFLP analysis [9] was used. DNA was extracted and RFLP typing was performed using the insertion sequence IS*6110* as a probe and *Pvu*II as the restriction enzyme. Visual bands were analyzed using the BioNumerics software version 5.10 (Applied Maths, Kortrijk, Belgium). Fingerprint patterns were compared by the un-weighted pair-group method of arithmetic averaging using the Jaccard coefficient. Dendrograms were constructed to show the degree of relatedness among strains according to a previously described algorithm [10], and similarity matrixes were generated to visualize the relatedness between the banding patterns of all isolates.

### PCR for RD702

Genomic DNA (10–20 ng) for RD702, which delineates the branch of *M. africanum* West African 2 lineage [11], [12] was amplified in a volume of 25 µL, which contained premixed *Taq,* Q solution (both from Qiagen), and primers RD702F (TTCCGAGGACCCGTTGTTGAGTGC) and RD702R (GGGCGGGTTGGGTTGCTGGTC) at a final concentration of 0.2 µmol/L [3], [11]. Conditions used for the amplification were as follows: 94°C for 3 min, followed by 35 cycles each of 94°C for 1 min, 64°C for 1 min, and 72°C for 1 min. PCR products of ∼ 2 kb for the control strain H37Rv and ∼ 700 bp for the isolates belonging to *M. africanum* West African 2 lineage were visualized on a 0.8% agarose gel [12].

### Single-nucleotide polymorphism in the *narGHJI* operon

A mutation in the *narGHJI* operon has been reported to be responsible for differential nitrate reductase activity of *M. tuberculosis* (-215T) versus *M. bovis* (-215C) [13]. The single-nucleotide polymorphism (SNP) at position -215 within the nitrate reductase (*narGHJI*) operon promoter in *M. tuberculosis* complex isolates with different NO_3_ activity was investigated. The isolates were characterized by PCR-RFLP using the previously published primers LC66 (AACCGACGGTGTGGTTGAC) and LC67 (ATCTCGATGGATGGGCGTC) [13]. The PCR products obtained were digested using the restriction endonuclease Sau3AI (Roche-Biomedicals, Meylan, France). This enzyme specifically cuts at the GATC sequence overlapping the *M. bovis*-like -215C *narGHJI* promoter site, producing two bands in PCR-RFLP of the LC66-LC67 PCR fragment. In the case of the -215T sequence, the restriction site is absent and as a result the PCR-RFLP of the *narGHJI* amplicon yields a single band.

### Geographical distribution of spoligotypes and lineages

The distribution of spoligotypes in the SITVIT2 database, in relation to their geographical origin was recorded for regions representing ≥5% of a given SIT as compared to their total number in the SITVIT2 database. The various macro-geographical regions and sub-regions were defined according to the specifications of the United Nations (http://unstats.un.org/unsd/methods/m49/m49regin.htm). Correlation of spoligotype families and principal genetic groups (PGG) based on *KatG463-gyrA95* polymorphisms [14] was inferred from the reported linking of specific spoligotype patterns to PGG1 (ancient lineages), and PGG2/3 (modern lineages) as previously described [8], [15], [16].

For statistical analysis of trends over time the Chi square test for trend was performed using PASW v 18.

## Results

In study A, spoligotyping and RFLP was performed on 229 isolates. In study B, adequate amounts of DNA, in order to perform RFLP, were retrieved from 132 isolates, while it was possible to perform spoligotyping on 138 isolates. In study C, spoligotyping could be performed on 47 isolates, while sufficient amounts of DNA were available for RFLP typing of 41 isolates. In regard to the remaining positive samples they were either too contaminated or the isolates had died during transportation. All isolates that were subjected to RFLP were also spoligotyped. For single-nucleotide polymorphism in the *narGHJI* operon, 28 isolates were selected from RFLP cluster B:5 [1] in study A.

### Spoligotyping

Altogether, 414 isolates were available for spoligotyping, of which 336 isolates were grouped into 42 clusters (2–96 isolates per cluster), accounting for a very high clustering rate of 81.2%, while the remaining 78 (18. 8%) strains did not cluster. Seven clusters included more than ten isolates each.

#### Study A

Out of 229 isolates, 82 different spoligotypes were obtained, of which 168 (73.4%) were clustered into 21 spoligo-clusters, comprising 2–61 isolates per cluster. The remaining 61 (26.6%) spoligopatterns were unique i.e. the isolates did not cluster with other patient isolates. The 229 isolates are identical to those reported in [1], however a mistake in the recording of one spoligotype (isolate IH-218) has been corrected here. Also, nine isolates with unique spoligotypes (summarized in Table 2 of reference [1]), were not mentioned in the Results section of the 1999 paper.

#### Study B

Of 138 isolates, 56 different spoligotypes were obtained, of which 98 (71.0%) were clustered into 15 spoligo-clusters, comprising 2–26 isolates per cluster. The remaining 40 (29.0%) spoligopatterns were unique i.e. the isolates did not cluster with other patient isolates.

#### Study C

Of 47 isolates, 26 different spoligotypes were obtained, of which 29 (61.7%) were clustered into 8 spoligo-clusters, comprising 2–9 isolates per cluster. The remaining 18 (38.3%) spoligopatterns were unique i.e. the isolates did not cluster with other patient isolates.

The spoligotyping results from the SITVIT2 database comparison are summarized in **Table 1**. Of 120 different patterns for the 414 strains studied 50 patterns corresponded to orphan strains that were unique among the 75,000 strains recorded in the SITVIT2 database (Supplementary **Table S1)**, as opposed to 70 patterns corresponding to 364 clinical isolates that corresponded to shared-types (Supplementary **Table S2**), i.e. an identical pattern shared by two or more patients worldwide (within this study, or matching another strain in the SITVIT2 database). A SIT number was attributed to each pattern according to the SITVIT2 database.

**Table 1:** Sublineage distribution and associated SIT designations for each of the lineages in the study.

Lineage	Sublineage	SIT
AFRI n = 195 (47.10%)	AFRI_1 n = 194	***SIT181*** n = 96
		SIT187 n = 46
		orphan n = 26
		SIT536 n = 6
		SIT188 n = 3
		SIT318 n = 2
		SIT326 n = 2
		SIT525 n = 2
		SIT530 n = 2
		SIT532 n = 2
		SIT3104 n = 2
		SIT3118* n = 2
		SIT324 n = 1
		SIT537 n = 1
		SIT3130 n = 1
	AFRI_3 n = 1	orphan n = 1
LAM n = 75 (18.12%)	LAM9 n = 43	***SIT42*** n = 36
		SIT2201 n = 2
		SIT3020* n = 2
		SIT766 n = 1
		SIT866 n = 1
		orphan n = 1
	LAM1 n = 14	SIT20 n = 11
		SIT534 n = 3
	LAM4 n = 9	***SIT60*** n = 7
		SIT828 n = 1
		orphan n = 1
	LAM5 n = 3	***SIT93*** n = 1
		orphan n = 2
	LAM2 n = 2	SIT604 n = 2
	LAM3 n = 2	SIT4 n = 1
		SIT3101* n = 1
	LAM10-CAM n = 2	***SIT61*** n = 2
T clade n = 53 (12.80%)	T1 n = 41	***SIT53*** n = 10
		SIT244 n = 7
		SIT334 n = 3
		SIT804 n = 3
		orphan n = 4
		SIT3100* n = 3
		SIT196 n = 2
		SIT522 n = 2
		SIT535 n = 2
		SIT230 n = 1
		SIT521 n = 1
		SIT611 n = 1
		SIT801 n = 1
		SIT888 n = 1
	T n = 8	SIT73 n = 5
		SIT3129* n = 2
		SIT2440 n = 1
	T5 n = 3	orphan n = 2
		***SIT44*** n = 1
	T2 n = 1	orphan n = 1
Haarlem n = 37 (8.94%)	H1 n = 22	***SIT47*** n = 18
		SIT62 n = 1
		SIT531 n = 1
		SIT2030* n = 1
		orphan n = 1
	H3 n = 15	***SIT50*** n = 12
		SIT533 n = 2
		SIT75 n = 1
EAI n = 25 (6.04%)	EAI5 n = 20	SIT527 n = 9
		SIT528 n = 4
		orphan n = 4
		SIT458 n = 2
		***SIT236*** n = 1
	EAI-SOM n = 4	SIT529 n = 3
		SIT538 n = 1
	EAI6-BGD n = 1	SIT129 n = 1
Unknown signatures n = 13 (3.14%)	Unk n = 13	orphan n = 6
		SIT2445 n = 2
		SIT3131* n = 2
		SIT523 n = 1
		SIT1200 n = 1
		SIT1204 n = 1
Beijing n = 7 (1.69%)	Beijing n = 7	***SIT1*** n = 7
× clade n = 4 (0.97%)	×3 n = 2	***SIT92*** n = 2
	×1 n = 2	***SIT119*** n = 2
		
Manu n = 4 (0.97%)	Manu2 n = 2	***SIT54*** n = 1
		orphan n = 1
	Manu1 n = 1	SIT3132* n = 1
	Manu-ancestor n = 1	***SIT523*** n = 1
CAS n = 2 (0.48%)	CAS1-DEL n = 2	SIT3111 n = 2

Newly-defined SITs are shown by an asterisk while the prototypes defining a sublineage in SITVIT2 database are shown in ***bold italics***.

When the overall repartition of strains according to major *M. tuberculosis* genotypic families was defined (**Table 1**), the major circulating *M. tuberculosis* clades ranked in the following order: AFRI (n = 195, 47.10%) > LAM (n = 75, 18.12%) > ill-defined T clade (n = 53, 12.80%) > Haarlem (n = 37, 8.85%) > EAI (n = 25, 6.04%) > Unknown (n = 12, 2.87%) > Beijing (n = 7, 1.68%) > X clade (n = 4, 0.96%) > Manu (n = 4, 0.97%), CAS (n = 2, 0.48%). Two strains of the LAM clade, both isolated in 2007, belonged to the Cameroon family (SIT61) [17].

The most predominant lineage in Guinea-Bissau was AFRI, which accounted for as high as 47.10% of all TB cases. Surprisingly all the cases with AFRI with the exception of a single orphan isolate classified as AFRI_3, were caused by the AFRI_1 sublineage split into two spoligopatterns – SIT181 (the prototype of the AFRI_1 sublineage) and SIT187. As compared to the SITVIT2, these two SITs were highly predominant in Guinea-Bissau since they alone accounted for 36.92% and 52.87% of all such strains in the database (414 of 423 isolates from Guinea-Bissau in the database originating from this study) with extremely high phylogeographical specificity for Western Africa (74.62 and 74.71% respectively, (Supplementary **Table S3**).

Another interesting observation was a pattern belonging to the EAI5 sublineage (SIT527, n = 9 strains in this study), with 100% of the cases being identified so far exclusively in Guinea-Bissau. These lineages are characteristic of the evolutionary ancient PGG1 strains. The remaining predominant SITs (SIT42 and SIT20 belonging to the LAM9 and LAM1 sublineages of LAM clade, and SIT47 and SIT50 belonging to the H1 and H3 sublineages of Haarlem clade) were characteristic of the evolutionary-recent PGG2/3 subgroups. The last predominant spoligotype (SIT53) also belonged to the evolutionary-recent T1 sublineage, nonetheless, the “T” family does not represent a genotypic lineage in a strict evolutionary sense since it was defined by default to include strains that could not be classified in one of the established lineages with well-established phylogeographical specificity such as the Haarlem, LAM, CAS, and EAI lineages [8].

Thus if one adds all the PGG1 isolates in Guinea-Bissau, i.e., AFRI n = 195, EAI n = 25, Beijing n = 7, Manu n = 4, CAS n = 2, and one unknown lineage pattern (SIT1200) that could be nonetheless classified as PGG1, the total number of ancestral strains in Guinea-Bissau amounts to 234/414 or 56.52% of all strains.

**Figure 1:**
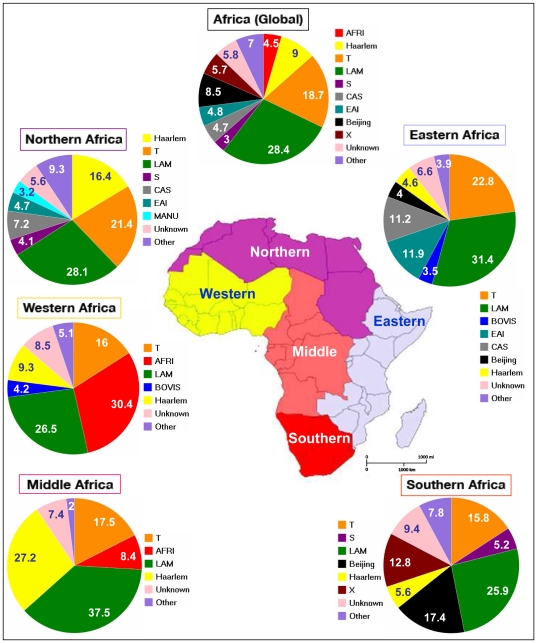
Geographical distribution of spoligotyping-based genotypic lineages of *Mycobacterium tuberculosis* in various subregions of Africa (n = 11956 clinical isolates) in the SITVIT2 database.

To get an indication of the distribution of the Guinea-Bissau family in Africa, and in spite of the fact that the SITVIT2 is not absolutely representative in terms of proportional representation, we decided to compare all the data obtained in Guinea-Bissau, and compared it to isolates in the SITVIT2 from various subregions of Africa following United Nations geoscheme, i.e., Eastern Africa, Middle Africa, Northern Africa, Southern Africa, and Western Africa (which included Guinea-Bissau). The results obtained for a global collection of 11956 clinical isolates in the database are summarized in **Figure 1**, and highlight major differences in the population structure of circulating tubercle bacilli in Africa. It should be noted that to facilitate comparison, results were only tabulated for all major lineages with a cut-off at 3% (i.e., strains belonging to all minor lineages below the cut-off 3% are pooled together and shown as “Other”). Furthermore, all spoligotyping signatures that are not yet associated to a well-defined genotypic lineage in SITVIT2 were designated as “Unknown”.

As for other subregions, this distribution in Guinea-Bissau does not take into account the minor lineages below the cut-off of 3% that included Beijing, X, Manu and CAS lineages.

### RFLP

Out of the 402 isolates investigated, 193 different RFLP patterns were obtained, of which 268 (66.7%) isolates were clustered into 61 RFLP-clusters, comprising 2–45 isolates per cluster. The remaining 132 (32.8%) RFLP patterns were unique i.e. the isolates did not cluster with other patient isolates. Of the RFLP-typed isolates with an *M. africanum* spoligotype 146/193 (75.6%) isolates, and 121/209 (57.9%) of the non-*M. africanum* isolates were clustered into 24 and 37 RFLP-clusters respectively. Of those with an *M. africanum* spoligotype 125/193 had 5 bands or less (median 5 bands) and 38/209 of the non-*M. africanum* isolates had 5 bands or less (median 9 bands).

### RD702

Of the 195 isolates with a *M. africanum* signature 192 isolates were analyzed for RD702; they all showed a PCR product of ∼ 700 bp indicating that RD702 is absent in the Guinea-Bissau family isolates.

### Single-nucleotide polymorphism in the *narGHJI* operon

Twenty-eight Guinea-Bissau isolates, representing all five biovars [4], were investigated. Six of the isolates were nitrate reductase positive, while 22 were negative. The *M. tuberculosis* H37Rv reference strain served as a control. All 28 isolates showed a single PCR product with a size of approximately 155 bp. For all but one of the Guinea-Bissau family strains, irrespective of nitrate reductase activity, the subsequent digestion with Sau3AI, specifically cutting at the GATC sequence, produced two bands (∼ 90 and ∼ 70 bp) showing that these strains harbored the -215C promoter SNP. For the *M. tuberculosis* H37Rv reference strain and one Guinea-Bissau strain (nitrate positive) cleavage was not successful, resulting in a single, uncut band of approximately 155 bp.

### Trends over time

The proportion of AFRI_1 significantly decreased over time when the three studies A, B and C were compared (P-value  = 0.048). Thus in study A 118 (52%) of 229 isolates were AFRI, in study B 60 (44%) of 138 isolates were AFRI, and in study C 18 (38%) of 47 isolates were AFRI. The AFRI_3 isolate was found in 1996 (study B).

## Discussion

**The Guinea-Bissau family is a member of the** ***M. africanum*** **West African 2 lineage** Traditionally, *M. africanum* strains were defined according to their phenotypic traits and geographic origin, and were accordingly named West African (subtype I) and East African (subtype II) *M. africanum* [4], [18]. Based on genetic definitions so called *M. africanum* subtype II strains are now regarded as phenotypic variants of *M. tuberculosis* [19], [20], [21]. *M. africanum* subtype I (West African *M. africanum*) strains are defined on the basis of particular genotypic characteristics that place them in the *M. tuberculosis* complex phylogenetic tree [2], [19], [22], [23]. Thus, genomic deletions, or regions of difference (RDs), can effectively distinguish them from *M. tuberculosis* and *M. bovis*. Isolates of West African *M. africanum* lack RD9 and are positive for RD12 [19], [20]. We subsequently refer to *M. africanum* subtype I as *M. africanum*. *M. africanum* strains can be further subdivided into two main lineages, West African 1 and West African 2 [12], [20]. West African 1 isolates lack RD711, while West African 2 isolates lack RD 7, 8, 10 and 702 [12], [24]. By spoligotyping *M. africanum* is defined by loss of spacers 8, 9 and 39. Three sublineages are defined based on finer differences: AFRI_1 by loss of spacers 7–9, and 39, AFRI_2 by loss of spacers 8–12, 21–24, and 37–39, and AFRI_3 by loss of spacers 8–12, and 37–39. These subdivisions correspond to LSP-defined classification as follows: AFRI_1 corresponds to West African 2, while AFRI_2 and AFRI_3 collectively constitute the West African 1 lineages.

This study of *M. tuberculosis* complex isolates from Guinea-Bissau, collected during two decades, shows that TB in Guinea-Bissau is essentially caused by predominant genotypes characteristic of the Western and Middle African region, with the highest prevalence of *M. africanum* (AFRI_1/West African 2) in the whole African continent. Forty-seven percent of all isolates were of what we call the Guinea-Bissau family, a member of the *M. africanum* AFRI_1/West African 2 lineage.

Thus, they have an AFRI_1 spoligotype pattern [1] (this study), they have a distinct RFLP pattern with low numbers of IS*6110* insertions [1] (this study) and they lack the four regions of difference RD7, RD8, RD9 and RD10 [2], as well as RD701 [3] and RD702 (this study). This profile classifies the Guinea-Bissau family strains, irrespective of phenotypic biovar, as part of the *M. africanum* West African 2 lineage [24] or the AFRI_1 sublineage according to the spoligotyping nomenclature. This family shows an extremely high phylogeographical specificity for Western Africa, with Guinea-Bissau being the epicenter.

**Other lineages.** In addition to the AFRI isolates 25 isolates were of the “ancient” EAI lineage, and the remaining strains were “modern” *M. tuberculosis* strains. These latter strains most likely represent strain importation from countries where such strains predominate. Only a few isolates were of the Beijing lineage, which is relatively rare in Africa, except for parts of South Africa [25].

Historically, Guinea-Bissau is a former colony of Portugal, a country with a high incidence of TB caused by the classical human *M. tuberculosis* type. Thus, contacts between the populations of Guinea-Bissau and Portugal have probably been more numerous than contacts between the populations of Guinea-Bissau and other African countries. We therefore speculate that these *M. tuberculosis* strains may have their origin from within Portugal [1]. Indeed, some of the isolates had RFLP patterns that were identical to isolates from Portuguese TB patients [1].

An interesting observation was the exclusive presence of an EAI5 sublineage (SIT527) in Guinea-Bissau. LAM10-CAM strains were found in a very low proportion, with only 2/414 (0.5%) of such isolates, interestingly both strains belonged to LAM10-CAM prototype SIT61. Yet they are predominant in Middle Africa and Western Africa, with phylogeographical specificity of LAM10-CAM for Cameroon and neighboring countries in West Africa [8], [26], and a recent study showed that LAM10-CAM prototype SIT61 was also the most predominant spoligotype in Cotonou, Benin [27].

Another interesting feature was the total absence of *M. bovis* in our dataset. Similarly, the finding of four Manu strains in Guinea-Bissau is noteworthy. The "Manu" lineage was initially described as a new family from India in 2004 [28], and later similar strains in small numbers were reported in a study from Madagascar [29]. Soon afterwards, it was tentatively subdivided into Manu-1 (deletion of spacer 34), Manu-2 (deletion of spacers 33–34) and Manu-3 (deletion of spacers 34–36) sub-lineages, with the suggestion that it could represent an ancestral clone of PGG1 strains [8]. More recently, Manu lineage strains were reported from Saudi Arabia, [30] Tunisia [31], and Egypt [32]. In the latter study, Manu lineage strains represented as high as 27% of all isolates, with the new addition of SIT523 (all 43 spacers present) designated as “Manu-ancestor” [32]. However, it was recently suggested that some Manu strains may eventually correspond to a “mixed” pattern due to concomitant Beijing and Euro-American lineage strains (the latter comprising H, LAM, X, and T lineages in spoligotyping defined clades), a possibility that may be investigated by RD105 analysis of Manu pattern isolates [33].

### Phenotype and genotype

Although the *M. africanum-*specific deletions each have putative biological consequence, they do not directly account for these mentioned variable biochemical characteristics. However, genomic variations other than the deletion of large sequence polymorphisms (LSPs) may be relevant for the observed phenotypic variability. Small polymorphisms, single nucleotide polymporhisms (SNPs), gene rearrangements, or genomic regions absent from H37Rv but present in *M. africanum* (duplications, insertions) are overlooked when applying RD analysis. As a result, phenotypic heterogeneity among *M. africanum* may be due to unidentified genetic polymorphisms.

Classical *M. bovis* strains, in contrast to *M. tuberculosis* strains, are naturally resistant to PZA and lack PZase activity. A point mutation in the *pncA* gene has been thought to be the cause of the defective PZase activity and PZA resistance in classical *M. bo*vis strains [34]. We have investigated isolates of the Guinea-Bissau family (Study A), and found that irrespective of phenotypic PZase activity, they all had the *M. tuberculosis* genotype in the *pncA* gene [1] (data not shown). This was true also for isolates lacking PZase activity, indicating that the variation in phenotypic PZase activity is not due to a variation in the *pncA* gene.

The differential nitrate reductase (NO_3_) activity of *M. tuberculosis* versus *M. bovis* has been reported to be due to a SNP in the *narGHJI* operon promoter [13]. The Guinea-Bissau strains tested, except for one, all carried the -215C *narGHJI* promoter genotype, as defined by the two-band pattern observed in PCR-RFLP. This genotype is present in *M. bovis* and some ancestral *M. tuberculosis* [35], and has been connected with a reduced nitrate reductase production. Expression of *narGHJI* is typically induced under anaerobic conditions, anaerobic nitrate reductase activity being expressed in both -215T and -215C genotypes. However, anaerobic nitrite reductase activity is considerably lower in *M. bovis*, which carries the -215C genotype, compared to *M. tuberculosis*. Diagnostic nitrate reductase activity is generally measured as the rapid accumulation of nitrite by the bacilli within two hours. *M. bovis* typically needs a longer time to accumulate detectable amounts of nitrite from nitrate [13]. Thus, the variable expression of nitrate reductase by *M. africanum* may be due to the narrow time-detection limit of nitrite defined by the test used and not a genotypic difference between individual *M. africanum* strains.

The differential expression of these biochemical traits is therefore not related to any genes that have been reported to differentiate between *M. bovis* and *M. tuberculosis*. Instead, we hypothesize that Guinea-Bissau strains may more readily switch genes on and off during its adaptation to hosts of different species (and hence broaden their host tropism) [4].

### Will the Guinea-Bissau family of strains disappear from West Africa?

This analysis shows that there exists a clear gradient in the African continent; *M. africanum* being predominant in Western and Middle Africa, EAI and CAS in Northern and Eastern Africa, Beijing and X essentially limited to Southern Africa, while LAM, Haarlem, and T are distributed throughout.

During the 15-year period since isolates were first collected, the proportion of isolates of the Guinea-Bissau family have diminished over time. This is in agreement with trends in some other West African countries, Cameroon [17] and Burkina Faso [36], where the proportion of *M. africanum* isolates have drastically decreased during the last decades. *M. africanum* appears at various frequencies in West Africa. In Sierra Leone a recent study identified 23/97 (24%) isolates as *M. africanum* [37], which appears to be similar to that reported from a study in 1992/1993 [18]. In The Gambia, 38% of isolates were *M. africanum* [38] and in Ghana 29% [39]. In the 1970's, most cases of TB in Cameroon were caused by *M. africanum* [40], while three decades later only 9% of cases were reported to be caused by *M. africanum* [17]. This change appears to be due to the recent expansion of the so-called Cameroon family of *M. tuberculosis* [17], [26]. In Burkina Faso, *M. africanum* was not detected in a recent survey [36] although *M. africanum* strains (defined by phenotypic characteristics) were reported in about 20% of cases two decades earlier [36], [41], [42]. Interestingly, during 2007, two strains of the Cameroon family had found their way into our setting.

The reason for the relative decline of *M. africanum* in West Africa could be a lower transmission capacity or virulence. There is a considerable uncertainty about the virulence of *M. africanum*. In studies from The Gambia [11], [38], [43], [44] and Ghana [39] this issue has been addressed. No difference in virulence, as assessed by the severity of radiological presentation, was found between the West African 1 and West African 2, and *M. africanum* was seen to closely resemble *M. tuberculosis* in pathology [39]. The pathology in patients with smear positive TB caused by *M. africanum* (West African 2) were found to be as severe as that of *M. tuberculosis* patients [44]. However, when comparing *M. tuberculosis* and *M. africanum* (West African 2) the rate of progression to disease was higher in household contacts of index patients with *M. tuberculosis* compared to those with *M. africanum* infection [43]. Interestingly, *M. africanum* elicited an attenuated T cell response to early secreted antigenic targets in patients with TB and their household contacts [11].

Another reason for the decline of *M. africanum* in West Africa could be that certain “modern” lineages possess advantages in their ability to disseminate within a community, in relation to more “ancient” lineages such as *M. africanum*. Of particular interest in this context is the appearance of Beijing isolates, as well as the recent isolation of two strains of the Cameroon family in our study. Beijing strains have recently been shown to have emerged and rapidly expanded in South Africa [25].

In conclusion the Guinea-Bissau family of strains, irrespective of phenotypic biovar, is part of the *M. africanum* West African 2 lineage, or the AFRI_1sublineage according to the spoligotyping nomenclature. The variation of biochemical traits classically used to differentiate *M. tuberculosis* from *M. bovis* is not related to any genes so far investigated (*narGHJI* and *pncA*). The Guinea-Bissau family shows an extremely high phylogeographical specificity for Western Africa, with Guinea-Bissau being the epicenter. Trends over time however indicate that this family of strains is waning in most parts of Africa, including Guinea-Bissau.

## Supporting Information

Table S1 Description of the orphan strains (n = 50) and corresponding spoligotyping defined lineages found among a total of 414 *M. tuberculosis* clinical isolates from studies performed in Guinea Bissau.
(DOC)

Table S2 Description of 70 shared types containing 364 *M. tuberculosis* complex clinical isolates from Guinea Bissau (GNB).
(DOC)

Table S3 Description of predominant SITs (representing 10 or more strains) in Guinea Bissau (GNB), and their worldwide distribution.
(DOC)
