# Supplementary material for: The Guinea-Bissau Family of Mycobacterium tuberculosis Complex Revisited
Source: PLoS One. 2011 Apr 20;6(4):e18601. doi: 10.1371/journal.pone.0018601 (PMC3080393; doi:10.1371/journal.pone.0018601)
Supplement: Table S1 — Description of the orphan strains (n = 50) and corresponding spoligotyping defined lineages found among a total of 414 M. tuberculosis clinical isolates from studies performed in Guinea Bissau. (DOC) [file pone.0018601.s001.doc]

**Table S1.** Description of the orphan strains (n=50) and corresponding spoligotyping defined lineages found among a total of 414 *M. tuberculosis* clinical isolates from studies performed in Guinea Bissau.

| ISO Number ^a^ | Year | Strain | Spoligotype Description | Octal code | Clade ^b^ |
| --- | --- | --- | --- | --- | --- |
| GNB0119891G-23/89 | 1989 | G-23/89 | ■□■■■■■■■■■■□□□□■■■■□□□□■■■■■■■■□□□□■■■■■■■ | 577703607760771 | LAM 5 |
| GNB0119891G-26/89 | 1989 | G-26/89 | ■□□■■■■■■■■■■■■■■□■■□□■■□□□□□□□□□□□□□□□□□□□ | 477776630000000 | Unk |
| GNB0119891G-31/89 | 1989 | G-31/89 | ■□■■■■■■■■■■■■■■■■■■□□■■■□□□□□□■□□□□■■■■■■■ | 577777634020771 | H1 |
| GNB0119891G-35/89 | 1989 | G-35/89 | ■□■■■■■■■■■■■■■■■■■■□□□□■■■■■■■□□□□□■■■□■■■ | 577777607740731 | LAM 4 |
| GNB0119891G-55/89 | 1989 | G-55/89 | ■□■■■■■■■■■■■□□□□□■□■□□■■■■■■■■□□□□□■■■■■■■ | 577740517740771 | Unk |
| GNB0119901G-103/90 | 1990 | G-103/90 | ■□■■■■■■■■■■■□□■■■■■□□□□■■■■■■■■□□□□■■■■■■■ | 577747607760771 | LAM 9 |
| GNB0119901G-16/90 | 1990 | G-16/90 | ■■■■■■□□□■■■□□□□■■□□■□□□■■■■■■■■□■■■■■□■■■■ | 770703107767671 | AFRI_1 |
| GNB0119901G-31/90 | 1990 | G-31/90 | ■□■■■■■■■■■■■■■■■■■■■■■■■□□□□□□□□□□□■■■■■■■ | 577777774000771 | Unk |
| GNB0119901G-37/90 | 1990 | G-37/90 | ■□■■■■□□□■■■■■■■■■■■■■■■■■■■■□□□□□□□□□□■■■■ | 570777777600071 | Unk |
| GNB0119901G-42/90 | 1990 | G-42/90 | ■■■■■■□□□■■■■■■■■■■■□□■■■■■■■■■□□□■■■■□■■■■ | 770777637743671 | AFRI_1 |
| GNB0119901G-72/90 | 1990 | G-72/90 | ■■■■■■□□□■■■■■■■■■■■■■■■■■■■■■■□□□■■■■□■■■■ | 770777777743671 | AFRI_1 |
| GNB0119921IH-112 | 1992 | IH-112 | ■■□■■■■■■■■■■■■■■■■■■■■■■■■■□□□□■□■■■■□□■■■ | 677777777413631 | EAI 5 |
| GNB0119921IH-143 | 1992 | IH-143 | ■■■■■□□□□□□□□□□□□□□□□■■■■■■■■■■■■■■■■■□■■■■ | 760000077777671 | AFRI_1 |
| GNB0119921IH-148 | 1992 | IH-148 | ■■■■■■□□□■■■■■■■■■■■■■■■■■■□□□□□□□□□□□□□□□□ | 770777777000000 | Unk |
| GNB0119921IH-165 | 1992 | IH-165 | ■■■■■■■■■■■■■■□■■■■■■■■■■■■■□■■■□□□□■■■□■■■ | 777767777560731 | T2 |
| GNB0119921IH-176 | 1992 | IH-176 | ■■■■■■■■■■■■■■■■■■■■□□■■■■■■■■■■□□■■■■■■■■■ | 777777637763771 | Manu 2 |
| GNB0119921IH-203 | 1992 | IH-203 | ■■■■□□□□□□□□□■■■■■■■■■■■■■■■■■■■■■■■■■□■■■■ | 740037777777671 | AFRI_1 |
| GNB0119921IH-270 | 1992 | IH-270 | ■■□■■■■■■■■■■■■■■■■■■■■■■■■□□□□□□□■■■■■■■■■ | 677777777003771 | EAI 5 |
| GNB0119923IH-32 | 1992 | IH-32 | ■■■■■□□□□□■■■■■■■■□■■■■■■■■■■■■□□□■■■■□■■■■ | 760377377743671 | AFRI_1 |
| GNB0119921IH-40 | 1992 | IH-40 | ■■■■■□□□□□■■■■■■■■■■■■□■■■■■■■■■■■■■■■□■■■■ | 760377757777671 | AFRI_1 |
| GNB0119923IH-49 | 1992 | IH-49 | ■■□■■■□□□■■■■■■■■■■■■■■■■■■■■■■■■■■■■■□■■■■ | 670777777777671 | AFRI_1 |
| GNB0119921IH-51 | 1992 | IH-51 | ■■■□■■■■■■■■■■□■■■■■■■■■■■■■■■■■□□□□■■■■■■■ | 737767777760771 | T1 |
| GNB0119921IH-68 | 1992 | IH-68 | ■■■■■■■■■■□□□□□■■■■■□□□□■■■■■■■■□□□□■■■■■■■ | 777407607760771 | LAM 5 |
| GNB0119921IH-76 | 1992 | IH-76 | ■■■■■■□■□■■□□□□■■■■■■■■■■■■■■■■■■■■■■■□■■■■ | 772607777777671 | AFRI_1 |
| GNB0119921IH-77 | 1992 | IH-77 | ■■■■■■□□□■■■■■■■■■■■■■■■■□□■■■■■■■■■■■□■■■■ | 770777774777671 | AFRI_1 |
| GNB0119921IH-84 | 1992 | IH-84 | ■■■■■■■□■■■■■■■■■■■■□■□■□■■■■■■■□□□□■■■■■■■ | 775777653760771 | T5 |
| GNB0119921IH-85 | 1992 | IH-85 | ■■■■■■□□□■■■■■■■■■■■■□□□■■■■■■■■■■■□■■□■■■■ | 770777707776671 | AFRI_1 |
| GNB0119921SN-139 | 1992 | SN-139 | ■■■■■■□□□■■■■■■■■■■■■■■■■□■■■■■■□■■■■■□■■■■ | 770777775767671 | AFRI_1 |
| GNB0119921SN-154 | 1992 | SN-154 | ■■■■■■□□□■■■■■■■■■■■■■■■■■■■■■■■■■■■■■□□□□□ | 770777777777600 | AFRI_1 |
| GNB0119921SN-359 | 1992 | SN-359 | ■□□■■■□□□■■■■■■■■■■■■■■■■■■■■■■■■■■■■■□■■■■ | 470777777777671 | AFRI_1 |
| GNB0119931SN-379 | 1993 | SN-379 | ■■■■■■□□□■■■■■■■■■■■■■■■■■■■□□□□□□■■■■□■■■■ | 770777777403671 | AFRI_1 |
| GNB0119931SN-380 | 1993 | SN-380 | ■■■■■■□□□■■■■■□■■■■■■■■■■■■■□□□□□□■■■■□■■■■ | 770767777403671 | AFRI_1 |
| GNB0219941A-189 | 1994 | A-189 | ■■■■■■□□□□□□□□□□■■■■■■■■■■■■■■■■□■■■■■□■■■■ | 770003777767671 | AFRI_1 |
| GNB0219941A-201 | 1994 | A-201 | ■■■□■■□□□■■■■■■■■■■□□■■■□□□□□□□□□□□■■■□■■■■ | 730777470001671 | AFRI_1 |
| GNB0219941A-30 | 1994 | A-30 | ■□□■■■■■■■■■■■■■□■■■■■■■■■■■□□□□□□■■■■■■■■■ | 477775777403771 | EAI 5 |
| GNB0219961TBS-058 | 1996 | TBS-058 | ■■■■■■■■■■■■■■■■■■■□■□□□■■■■■■■■□□□□■■■■■■■ | 777777507760771 | T1 |
| GNB0219961TBS-077 | 1996 | TBS-077 | ■■■■■■■□□□□□■■■■□□□□□□□□■■□□□□□■■■□■□□□■■■■ | 774074006035071 | AFRI_3 |
| GNB0219961TBS-081 | 1996 | TBS-081 | ■□■■■■□□□■■■■■■■■■■■■■■■■■■■■■■■■■■■□■□■■■■ | 570777777777271 | AFRI_1 |
| GNB0219973TBS-250 | 1997 | TBS-250 | ■■□■■■■■■■■■■■■■■■■■■■■■■■■■□□□□□□■■■■■□□□■ | 677777777403701 | EAI 5 |
| GNB0219971TBS-275 | 1997 | TBS-275 | ■■■■■■□□□■■■■■□□□■■■■■■■■■■■■■■■■■■■■■□■■■■ | 770761777777671 | AFRI_1 |
| GNB0219973TBS-299 | 1997 | TBS-299 | ■■■■■■□□□■■■■■■■■■■■■■■■■■■■■■■■■■■■■■□□□■■ | 770777777777611 | AFRI_1 |
| GNB0219971TBS-355 | 1997 | TBS-355 | ■■■■■■■■■■■■■■■■■■■■■■□■■■■■■■■■□□□□■■■■■■□ | 777777757760770 | T5 |
| GNB0219971TBS-374 | 1997 | TBS-374 | ■■■■■■■■■■■■■■□□□■■■■■■■■■■■■■■■□□□□■■□□□□■ | 777761777760601 | T1 |
| GNB0219970TBS-381 | 1997 | TBS-381 | ■■■■■■□□□■■■■■■■■■■□□■■■■■■■■■■■■■■■■■□■■■■ | 770777477777671 | AFRI_1 |
| GNB0219980TBS-416 | 1998 | TBS-416 | □□□■■■□□□□■■■■■■■■■■■■■■■■■■■■■■□■■■■■□■■■■ | 070377777767671 | AFRI_1 |
| GNB0219980TBS-418 | 1998 | TBS-418 | ■■■■■■□□□□■■■■■□■■■■■■■■■■■■■■■■□■■■■■□■■■■ | 770373777767671 | AFRI_1 |
| GNB0220060XTB07-042 | 2006 | XTB07-042 | ■□■■■■□□□□□■■■■■■■■■□■■■■■■■■■■□□□□□■■□■■■■ | 570177677740671 | T1 |
| GNB0220060XTB07-051 | 2006 | XTB07-051 | ■□■■□■■■■□■■■■□■■■■■□■■■■■■■■■■■□□■■■■□■■■■ | 557367677763671 | AFRI_1 |
| GNB0220073XTB08-040 | 2007 | XTB08-040 | ■■■■■■■■■■□□□□■■■■■■■□□□□□□□□□□□□□□□□□□□□□□ | 777417700000000 | Unk |
| GNB0220071XTB08-080 | 2007 | XTB08-080 | ■■■■■■□□□■■■■■■■■■■■■■■■■■■■■■■■■■■■■■□■□■■ | 770777777777651 | AFRI_1 |

^a^ The ISO number in the SITVIT2 database linked to each individual strain data entered.

^b^ Clade designations according to SITVIT2 using revised SpolDB4 rules; Unk: Unknown patterns within any of the major clades described in SITVIT2.
