# Supplementary material for: The Guinea-Bissau Family of Mycobacterium tuberculosis Complex Revisited
Source: PLoS One. 2011 Apr 20;6(4):e18601. doi: 10.1371/journal.pone.0018601 (PMC3080393; doi:10.1371/journal.pone.0018601)
Supplement: Table S2 — Description of 70 shared types containing 364 M. tuberculosis complex clinical isolates from Guinea Bissau (GNB). (DOC) [file pone.0018601.s002.doc]

**Table S2.** Description of 70 shared types containing 364 *M. tuberculosis* complex clinical isolates from Guinea Bissau (GNB). A total of 60 SITs containing 347 isolates matched a preexisting shared type in the SITVIT2 database, whereas 10 SITs (containing 17 Isolates) were newly-created either within the present study or after a match with an orphan in the database.

| SIT ^a,b^ | Spoligotype Description | Octal code | Number (%) GNB | % in GNB compared to SITVIT2 | Clade ^c^ | Clustered vs. unique patterns ^d^ |
| --- | --- | --- | --- | --- | --- | --- |
| 1 | □□□□□□□□□□□□□□□□□□□□□□□□□□□□□□□□□□■■■■■■■■■ | 000000000003771 | 7(1.69) | 0.11 | Beijing | Clustered |
| 4 | □□□□□□□□□□□□□□□□□□□□□□□□■■■■■■■■□□□□■■■■■■■ | 000000007760771 | 1(0.24) | 0.36 | LAM 3 | Unique |
| 20 | ■■□■■■■■■■■■■■■■■■■■□□□□■■■■■■■■□□□□■■■■■■■ | 677777607760771 | 11(2.66) | 1.57 | LAM 1 | Clustered |
| 42 | ■■■■■■■■■■■■■■■■■■■■□□□□■■■■■■■■□□□□■■■■■■■ | 777777607760771 | 36(8.7) | 1.31 | LAM 9 | Clustered |
| 44 | ■■■■■■■■■■■■■■■■■■■■■■□■■■■■■■■■□□□□■■■■■■■ | 777777757760771 | 1(0.24) | 0.54 | T5 | Unique |
| 47 | ■■■■■■■■■■■■■■■■■■■■■■■■■□□□□□□■□□□□■■■■■■■ | 777777774020771 | 18(4.35) | 1.47 | H1 | Clustered |
| 50 | ■■■■■■■■■■■■■■■■■■■■■■■■■■■■■■□■□□□□■■■■■■■ | 777777777720771 | 12(2.9) | 0.43 | H3 | Clustered |
| 53 | ■■■■■■■■■■■■■■■■■■■■■■■■■■■■■■■■□□□□■■■■■■■ | 777777777760771 | 10(2.42) | 0.21 | T1 | Clustered |
| 54 | ■■■■■■■■■■■■■■■■■■■■■■■■■■■■■■■■□□■■■■■■■■■ | 777777777763771 | 1(0.24) | 0.60 | Manu 2 | Unique |
| 60 | ■■■■■■■■■■■■■■■■■■■■□□□□■■■■■■■■□□□□■■■□■■■ | 777777607760731 | 7(1.69) | 1.92 | LAM 4 | Clustered |
| 61 | ■■■■■■■■■■■■■■■■■■■■■■□□□■■■■■■■□□□□■■■■■■■ | 777777743760771 | 2(0.48) | 0.29 | LAM10-CAM | Clustered |
| 62 | ■■■■■■■■■■■■■■■■■■■■■■■■■□□□□□□■□□□□■■■□■■■ | 777777774020731 | 1(0.24) | 0.24 | H1 | Unique |
| 73 | ■■■■■■■■■■■■□■■■■■■■■■■■■■■■■■■■□□□□■■■□■■■ | 777737777760731 | 5(1.21) | 2.45 | T | Clustered |
| 75 | ■■■■■■■■■■■■■■□■■■■■■■■■■■■■■■□■□□□□■■■■■■■ | 777767777720771 | 1(0.24) | 2.04 | H3 | Unique |
| 92 | ■■■□□□□□□□□□■■■■■□■■■■■■■■■■■■■■□□□□■■■■■■■ | 700076777760771 | 2(0.48) | 0.49 | X3 | Clustered |
| 93 | ■■■■■■■■■■■■□■■■■■■■□□□□■■■■■■■■□□□□■■■■■■■ | 777737607760771 | 1(0.24) | 0.31 | LAM 5 | Unique |
| 119 | ■■■■■■■■■■■■■■■■■□■■■■■■■■■■■■■■□□□□■■■■■■■ | 777776777760771 | 2(0.48) | 0.20 | X1 | Clustered |
| 129 | ■■■□□□□□□■■■■■■■■■■■■■□□■■■■□□□□■□■■■■■■■■■ | 700777747413771 | 1(0.24) | 2.38 | EAI6-BGD1 | Unique |
| 181 | ■■■■■■□□□■■■■■■■■■■■■■■■■■■■■■■■■■■■■■□■■■■ | 770777777777671 | 96(23.19) | 36.92 | AFRI_1 | Clustered |
| 187 | ■■■■■■□□□■■■■■■■■■■■■■■■■■■■■■■■□■■■■■□■■■■ | 770777777767671 | 46(11.11) | 52.87 | AFRI_1 | Clustered |
| 188 * | ■■■■■■□□□■■■■■■■■■■■■■■■■■■■■■■■■■■■■■□□■■■ | 770777777777631 | 3(0.72) | 75 | AFRI_1 | Clustered |
| 196 | ■■□■■■■■■■■■■■■■■■■■■■■■■■■■■■■■□□□□■■■■■■■ | 677777777760771 | 2(0.48) | 3.08 | T1 | Clustered |
| 230 | ■■■■■■■■■■■■■■■■■■■■□□□□□■■■■■■■□□□□■■■■■■■ | 777777603760771 | 1(0.24) | 5.56 | T1 | Unique |
| 236 | ■■■■■■■■■■■■■■■■■■■■■■■■■■■■□□□□■□■■■■■■■■■ | 777777777413771 | 1(0.24) | 0.72 | EAI 5 | Unique |
| 244 | ■■■■■■■■■■■■■■■■■■■■■■■■■■■■■■■■□□□□■■□□□□■ | 777777777760601 | 7(1.69) | 6.86 | T1 | Clustered |
| 318 | ■□■■■■□□□■■■■■■■■■■■■■■■■■■■■■■■■■■■■■□■■■■ | 570777777777671 | 2(0.48) | 33.33 | AFRI_1 | Clustered |
| 324 | ■■■■■■□□□■■■■■□■■■■■■■■■■■■■■■■■■■■■■■□■■■■ | 770767777777671 | 1(0.24) | 16.67 | AFRI_1 | Unique |
| 326 | ■■■■■■□□□■■■■■■■■■■■■□□□■■■■■■■■■■■■■■□■■■■ | 770777707777671 | 2(0.48) | 4.35 | AFRI_1 | Clustered |
| 334 | ■□■■■■■■■■■■■■■■■■■■■■■■■■■■■■■■□□□□■■■■■■■ | 577777777760771 | 3(0.72) | 4.55 | T1 | Clustered |
| 458 | ■■■■■■■■■■■■■■■■■■■■■■■■■■■■□□□□□□■■■■■■■■■ | 777777777403771 | 2(0.48) | 8.70 | EAI 5 | Clustered |
| 521 | ■■■■■■■■■■■■■■■■■■■■■■■■■■■■■■■■□□□□■■□□□■■ | 777777777760611 | 1(0.24) | 4 | T1 | Unique |
| 522 | ■■■■■■■■■■■■■■■■■■■■■■■■■■■■■■■■□□□□■■■■■■□ | 777777777760770 | 2(0.48) | 14.29 | T1 | Clustered |
| 523 | ■■■■■■■■■■■■■■■■■■■■■■■■■■■■■■■■■■■■■■■■■■■ | 777777777777771 | 1(0.24) | 2.94 | Manu-ancestor | Unique |
| 525 * | ■□■■■■□□□■■■■■■■■■■■□□■■■■■■■■■□□□■■■■□■■■■ | 570777637743671 | 2(0.48) | 100 | AFRI_1 | Clustered |
| 527 * | ■■□■■■■■■■■■■■■■■■■■■■■■■■■■□□□□□□■■■■■■■■■ | 677777777403771 | 9(2.17) | 100 | EAI 5 | Clustered |
| 528 | ■■□■■■■■■■■■■■■■■■■■■■■■■■■■□□□□■□■■■■□■■■■ | 677777777413671 | 4(0.97) | 44.44 | EAI 5 | Clustered |
| 529 | ■■□■■■■■■■■■■■■■■■■■■■■■■■■■□□□□■□■■■■■□■■■ | 677777777413731 | 3(0.72) | 60 | EAI1-SOM | Clustered |
| 530 * | ■■■■■■□□□■■■■■■■■■■□□■■■■■■■■■■■■■□■■■□■■■■ | 770777477775671 | 2(0.48) | 100 | AFRI_1 | Clustered |
| 531 | ■■■□■■■■■■■■■■■■■■■■■■■■■□□□□□□■□□□□■■■■■■■ | 737777774020771 | 1(0.24) | 16.67 | H1 | Unique |
| 532 * | ■■■■■■□□□■■■■■■■■■■■■■■□■■■■■■■■■■■■■■□■■■■ | 770777767777671 | 2(0.48) | 100 | AFRI_1 | Clustered |
| 533 * | ■■■■■■■■■■□□□□□□□□■■■■■■■■■■■■□■□□□□■■■■■■■ | 777400777720771 | 2(0.48) | 100 | H3 | Clustered |
| 534 | ■■■■■■■■■■■■■■■■■■■■□□□□■■■■□□□□□□□□□□□□□□□ | 777777607400000 | 3(0.72) | 30 | LAM | Clustered |
| 535 | ■■■■■■■■■■■■■■■■■■■■■□□□■■■■■■■■□□□□■■■■■■■ | 777777707760771 | 2(0.48) | 3.45 | T1 | Clustered |
| 536 | ■■■□□□□□□□□□□□□□□□□■■■■■■■■■■■■■■■■□■■□■■■■ | 700000377776671 | 6(1.45) | 75 | AFRI_1 | Clustered |
| 537 * | ■■■■■■□□□■■■■■■■■■■■■■■■■■■■■■■■■■■■■■□□□□■ | 770777777777601 | 1(0.24) | 50 | AFRI_1 | Unique |
| 538 * | ■■■■■■■■■■■□□■■■■■■■■■■■■■■■□□□□■□■■■■■□■■■ | 777637777413731 | 1(0.24) | 50 | EAI1-SOM | Unique |
| 604 | ■■□■■■■■■■■■□■■■■■■■□□□□■■■■■■■■□□□□■■■■□■■ | 677737607760751 | 2(0.48) | 15.38 | LAM 2 | Clustered |
| 611 | ■■■■■■■■■■■■■■□■■■■■■■■■■■■■□■■■□□□□■■■■■■■ | 777767777560771 | 1(0.24) | 50 | T1 | Unique |
| 766 | ■■■■■■■■■■■■■■□□□■□□□□□□■■■■■■■■□□□□■■■■■■■ | 777761007760771 | 1(0.24) | 3.13 | LAM 9 | Unique |
| 801 | ■■■■■■■□□■■■■■■■■■■■■■■■■■■■■■■■□□□□■■■■■■■ | 774777777760771 | 1(0.24) | 16.67 | T1 | Unique |
| 804 | ■□□■■■■■■■■■■■■■■■■■■■■■■■■■■■■■□□□□■■■■■■■ | 477777777760771 | 3(0.72) | 15.79 | T1 | Clustered |
| 828 | □■■■■■■■■■■■■■■■■■■■□□□□■■■■■■■■□□□□■■■□■■■ | 377777607760731 | 1(0.24) | 5 | LAM 4 | Unique |
| 866 | ■□■■■■■■■■■■■■■■■■■■□□□□■■■■■■■■□□□□■■■■■■■ | 577777607760771 | 1(0.24) | 2.44 | LAM 9 | Unique |
| 888 | ■■■■■■■■■■■■■■■■■■■■■■■■■■■■■■■■□□□□■■□□■■■ | 777777777760631 | 1(0.24) | 8.33 | T1 | Unique |
| 1200 | ■■■□□□□■■■■■■■■■■■■■■■□□■■■■■■■■■■■■■■■■■■■ | 703777747777771 | 1(0.24) | 12.5 | Unk | Unique |
| 1204 | ■■■■■■■■■■■■■■■□□□□□□□□□□□□□□□□□□□□□■■■□■■■ | 777770000000731 | 1(0.24) | 14.29 | Unk | Unique |
| 2030 * | ■■■■■■■■■■■■■■■■■■■■■□□□■□□□□□□■□□□□■■■■□■■ | 777777704020751 | 1(0.24) | 50 | H1 | Unique |
| 2201 | □□■■■■■■■■■■■■■■■■■■□□□□■■■■■■■■□□□□■■■■■■■ | 177777607760771 | 2(0.48) | 33.33 | LAM 9 | Clustered |
| 2440 | ■■■■■■□□□■■■■■■■■■■■■■■■■■■■■■■□□□□□■■□■■■■ | 770777777740671 | 1(0.24) | 33.33 | T | Unique |
| 2445 | ■□□■■■■■■■■■■■■■■■■■■■■■□□□□□□□□□□□□□□□□□□□ | 477777770000000 | 2(0.48) | 50 | Unk | Clustered |
| 3020 ** | ■■■■■■■■■■■■■■■■■■■■□□□□■■■□■■■■□□□□■■■■■■■ | 777777607360771 | 2(0.48) | 66.67 | LAM 9 | Clustered |
| 3100 ** | ■■■■■■■■■■□□■■■■■■■■■■■■■■■■■■■■□□□□■■■□□■■ | 777477777760711 | 3(0.72) | 100 | T1 | Clustered |
| 3101 ** | ■■■■□■■■□□□■■■■■■■■■□□□□■■■■■■■■□□□□■■■■■■■ | 756177607760771 | 1(0.24) | 50 | LAM 3 | Unique |
| 3104 ** | ■■■■■□□□□□■■■■■■■■■■■■■■■■■■■■■□□□■■■■□■■■■ | 760377777743671 | 2(0.48) | 100 | AFRI_1 | Clustered |
| 3111 ** | ■■■□□□□■■■■■■■■■■■■■■□□□□□□□□□□□□□□□□□□□□□□ | 703777700000000 | 2(0.48) | 100 | CAS1-Delhi | Clustered |
| 3118 ** | ■■■■■□□□□□■■■■■■■■■■■■■■■■■■■■■■■■■■■■□■■■■ | 760377777777671 | 2(0.48) | 100 | AFRI_1 | Clustered |
| 3129 ** | ■□□■■■■■■■■■□■■■■■■■■■■■■■■■■■■■□□□□■■■□■■■ | 477737777760731 | 2(0.48) | 100 | T | Clustered |
| 3130 ** | ■■■■■■□□□■■■■■■■■■■■■■■■■■■□■■■■■■■■■■□■■■■ | 770777777377671 | 1(0.24) | 50 | AFRI_1 | Unique |
| 3131 ** | ■■■■■■■■■■■■■■■■■■■■■■■■■■■□□□□□□□□□□□□□□■■ | 777777777000011 | 2(0.48) | 100 | Unk | Clustered |
| 3132 ** | ■■■■■■■■■■■■■■□■■■■■□■■■■■■■■■■■■□■■■■□■■■■ | 777767677773671 | 1(0.24) | 50 | Manu 1 | Unique |

^a^ SITs followed by a single asterisk indicate preexisting GNB data in SITVIT2 with previously attributed SIT designations (prior to entry of a new data file). Hence despite the values being mentioned as 50, 75 or 100 % under the column *“% in GNB compared to SITVIT2”*, these SITs are not newly-created. Their full listing with subsequent matching patterns and respective country codes is as follows: SIT188 (GNB n1, GBR n1), SIT525 (GNB n2), SIT527 (GNB n9), SIT530 (GNB n2), SIT532 (GNB n2), SIT533 (GNB n2), SIT537 (GNB n1), SIT538 (GNB n1, USA n1), SIT2030 (GNB n1, ITA n1).

^b^ SITs followed by a double asterisk indicate "newly created shared-type" (n=10) due an unique strain from this study that matched an orphan in the database (SIT3101 that matched an orphan from Spain); or due to 2 or more strains belonging to an identical new pattern within this study (SITs 3020, 3100, 3104, 3111, 3118, 3129, 3130, 3131, 3132; marked as “clustered” under the column “Clustered vs. unique patterns”; note that 3 newly-created SITs 3020, 3130, 3132, further matched a preexisting orphan in the database reported from Colombia, The Netherlands, and Indonesia, respectively).

^c^ Clade designations according to SITVIT2 using revised SpolDB4 rules; Unk: Unknown patterns within any of the major clades described in SITVIT2

^d^ Clustered strains correspond to a similar spoligotype pattern shared by 2 or more strains “within this study”; as opposed to unique strains harboring a spoligotype pattern that does not match with another strain from this study. Unique strains matching a preexisting pattern in the SITVIT2 database are classified as SITs, whereas in case of no match, they are designated as “orphan” (see Table 1).
