# Supplementary material for: The Guinea-Bissau Family of Mycobacterium tuberculosis Complex Revisited
Source: PLoS One. 2011 Apr 20;6(4):e18601. doi: 10.1371/journal.pone.0018601 (PMC3080393; doi:10.1371/journal.pone.0018601)
Supplement: Table S3 — Description of predominant SITs (representing 10 or more strains) in Guinea Bissau (GNB), and their worldwide distribution. (DOC) [file pone.0018601.s003.doc]

**Table S3.** Description of predominant SITs (representing 10 or more strains) in Guinea Bissau (GNB), and their worldwide distribution.

| SIT (Octal Number) Clade  Spoligotype Description | Total (%) in GNB | % in GNB as compared to SITVIT2 | Distribution in Regions with ≥ 5% of a given SIT ^a^ | Distribution in Countries with ≥ 5% of a given SIT ^b^ |
| --- | --- | --- | --- | --- |
| 181 (770777777777671) AFRI_1  ■■■■■■□□□■■■■■■■■■■■■■■■■■■■■■■■■■■■■■□■■■■ | 96(23.19) | 36.92 | AFRI-W 74.23, AMER-N 8.08, EURO-W 7.69 | GNB 37.30, GMB 32.31, USA 0.08 |
| 187 (770777777767671) AFRI_1  ■■■■■■□□□■■■■■■■■■■■■■■■■■■■■■■■□■■■■■□■■■■ | 46(11.11) | 52.87 | AFRI-W 74.71, AMER-N 12.64, AFRI-N 5.75 | GNB 54.02, GMB 18.39, USA 12.64 |
| 42 (777777607760771) LAM 9  ■■■■■■■■■■■■■■■■■■■■□□□□■■■■■■■■□□□□■■■■■■■ | 36(8.7) | 1.31 | AMER-S 28.38, AMER-N 15.49, EURO-S 12.16, AFRI-N 10.48, EURO-W 6.68 | USA 14.50, BRA 9.82, MAR 8.62, COL 7.49, ITA 6.39 |
| 47 (777777774020771) H1  ■■■■■■■■■■■■■■■■■■■■■■■■■□□□□□□■□□□□■■■■■■■ | 18(4.35) | 1.47 | AMER-N 20.95, EURO-W 20.13, EURO-S 13.77, AMER-S 10.43, EURO-E 8.39 | USA 19.40, AUT 10.27, ITA 7.25, BRA 5.87 |
| 50 (777777777720771) H3  ■■■■■■■■■■■■■■■■■■■■■■■■■■■■■■□■□□□□■■■■■■■ | 12(2.9) | 0.43 | AMER-N 21.85, AMER-S 15.90, EURO-W 15.36, EURO-S 12.63, EURO-E 6.46, AFRI-S 5.17 | USA 21.31, AUT 7.39, ESP 6.60, ITA 5.27 |
| 20 (677777607760771) LAM 1  ■■□■■■■■■■■■■■■■■■■■□□□□■■■■■■■■□□□□■■■■■■■ | 11(2.66) | 1.57 | AMER-S 24.39, AMER-N 24.39, AFRI-S 12.69, EURO-S 11.26, EURO-W 8.13, CARI 6.13 | USA 22.68, BRA 14.12, NAM 8.84, PRT 6.99, VEN 5.99 |
| 53 (777777777760771) T1  ■■■■■■■■■■■■■■■■■■■■■■■■■■■■■■■■□□□□■■■■■■■ | 10(2.42)) | 0.21 | AMER-N 19.40, AMER-S 14.26, EURO-W 12.64, EURO-S 9.88, ASIA-W 8.57, AFRI-S 6.41 | USA 17.04, ZAF 6.27, ITA 5.06 |
| 527 (677777777403771) EAI5  ■■□■■■■■■■■■■■■■■■■■■■■■■■■■□□□□□□■■■■■■■■■ | 9(2.17) | 100.00 | AFRI-W 100.00 | GNB 100.00 |

^a^ Worldwide distribution is reported for regions with ≥5% of a given SITs as compared to their total number in the SITVIT2. The definition of macro-geographical regions and sub-regions (<http://unstats.un.org/unsd/methods/m49/m49regin.htm>) is according to the United Nations; Regions: AFRI (Africa), AMER (Americas), ASIA (Asia), EURO (Europe), and OCE (Oceania), subdivided in: E (Eastern), M (Middle), C (Central), N (Northern), S (Southern), SE (South-Eastern), and W (Western). Furthermore, CARIB (Caribbean) belongs to Americas, while Oceania is subdivided in 4 sub-regions, AUST (Australasia), MEL (Melanesia), MIC (Micronesia), and POLY (Polynesia). Note that in our classification scheme, Russia has been attributed a new sub-region by itself (Northern Asia) instead of including it among rest of the Eastern Europe. It reflects its geographical localization as well as due to the similarity of specific TB genotypes circulating in Russia (a majority of Beijing genotypes) with those prevalent in Central, Eastern and South-Eastern Asia.

^b^ The 3 letter country codes are according to <http://en.wikipedia.org/wiki/ISO_3166-1_alpha-3>; countrywide distribution is only shown for SITs with ≥5% of a given SITs as compared to their total number in the SITVIT2 database.
